# Global world, global hospitals. Ethnic differences and psychotic symptoms presentation – a review

**DOI:** 10.1192/j.eurpsy.2022.1622

**Published:** 2022-09-01

**Authors:** A. Lourenço, M. Ribeiro, M. Lemos, A. Duarte, A. Neves

**Affiliations:** Centro Hospitalar Lisboa Norte, Psychiatry, Lisboa, Portugal

**Keywords:** ethnic differences, psychotic symptoms

## Abstract

**Introduction:**

We live in a global world, where immigration is no longer just an escape, but also a demand and a desire. Globalization imposes the challenge of recognizing psychiatric illness in the most diverse of patients.

**Objectives:**

To review the literature about the documentation of ethnic differences and the psychotic symptoms presentation.

**Methods:**

We performed a MEDLINE search using the key words: ethnic differences and psychotic symptoms. We only included studies with full text published in English.

**Results:**

Since the 1970s, some studies have shown that there are differences in the manifestation of psychiatric illness in ethnic minorities. Most recent studies confirm this statement, mainly with an increase in immigration in the 20th century, with the receiving countries having an increase in the number of cases of psychosis (affective and non-affective). Belonging to an ethnic minority increases the risk of psychotic symptoms and experiences, witch is related to the patients perception of discrimination, social differences, family separation and the stress associated with immigration. On the other hand, these groups also have less access to health care.

**Conclusions:**

Currently, professionals are more aware of the global world and what this implies in the manifestations of psychiatric illnesses. However, more studies will be needed to identify these natural differences. In this way, we will be able to help our patients anywhere and support their families.

**Disclosure:**

No significant relationships.

